# Biochar Amendment and Nitrogen Fertilizer Contribute to the Changes in Soil Properties and Microbial Communities in a Paddy Field

**DOI:** 10.3389/fmicb.2022.834751

**Published:** 2022-03-23

**Authors:** Izhar Ali, Pengli Yuan, Saif Ullah, Anas Iqbal, Quan Zhao, He Liang, Abdullah Khan, Imran, Hua Zhang, Xiaoyan Wu, Shanqing Wei, Minghua Gu, Ligeng Jiang

**Affiliations:** ^1^College of Agriculture, Guangxi University, Nanning, China; ^2^College of Life Science and Technology, Guangxi University, Nanning, China; ^3^Department of Agronomy, Faculty of Plant Sciences, The University of Agriculture, Peshawar, Pakistan

**Keywords:** soil properties, biochar, soil fungi and bacteria, rice rhizosphere, grain yield

## Abstract

Biochar amendment can influence the abundance, activity, and community structure of soil microbes. However, scare information is present about the effect of the combined application of biochar with synthetic nitrogen (N) fertilizer under paddy field condition. We aimed to resolve this research gap in rice field conditions through different biochar in combination with N fertilizers on soil nutrients, soil microbial communities, and rice grain yield. The present study involves eight treatments in the form of biochar (0, 10, 20, and 30 t ha^–1^) and N (135 and 180 kg ha^–1^) fertilizer amendments. The soil microbial communities were characterized using high-throughput sequencing of 16S and Internal transcribed spacer (ITS) ribosomal RNA gene amplicons. Experiential findings showed that the treatments had biochar amendments along with N fertilizer significantly advanced soil pH, soil organic carbon (SOC), total nitrogen (TN), soil microbial carbon (SMBC), soil microbial nitrogen (SMBN), and rice grain yield in comparison to sole N application. Furthermore, in comparison with control in the first year (2019), biochar amendment mixed with N fertilizer had more desirable relative abundance of microorganism, phyla Acidobacteria, Actinobacteria, Proteobacteria, and Verrucomicrobia with better relative abundance ranging from 8.49, 4.60, 46.30, and 1.51% in T7, respectively. Similarly, during 2020, bacteria phyla Acidobacteria, Actinobacteria, Bacteroidetes, Gemmatimonadetes, Planctomycetes, and Verrucomicrobia were resulted in higher and ranging from 8.69, 5.18, 3.5, 1.9, 4.0, and 1.6%, in biochar applied treatments, respectively, as compared to control (T1). Among the treatments, *Sphingopyxis* and *Thiobacillus* bacterial genus were in higher proportion in T7 and T3, respectively, as compared to other treatments and *Bacillus* was higher in T6. Interestingly, biochar addition significantly decreased the soil fungi phyla Ascomycota, Basidiomycota, Chytridiomycota, and Rozellomycota, in 2020 as compared to 2019. Whereas biochar addition to soil decreased *Echria*, *Kohlmeyeriopsis*, and *Westerdykella* fungal genus as compared to non-biochar treatments. The redundancy analysis showed that soil biochemical traits were positively correlated with soil bacteria. In addition, correlation analysis showed that soil bacteria including Acidobacteria, Actinobacteria, Bacteroidetes, Planctomycetes, and Proteobacteria strongly correlated with rice grain yield. This study demonstrated that soil nutrients and bacteria contribute to an increase in rice yield in combined biochar amendment with lower N treatments.

## Introduction

Soil microorganisms play an important role in the soil ecosystem, which are important for soil quality and agricultural productivity. They have an impact on several critical and fundamental ecosystem processes, such as organic matter decomposition, nutrient mineralization, soil functionality, and plant nutrient uptake and growth (Bending et al., 2004; Khan et al., 2021). Soil management practices directly affect the abundance and structure of soil microbes (Li et al., 2012). Furthermore, bacteria are the most diverse and abundant group of soil microorganisms, which play a significant role in the decomposition and mineralization of organic matter and nutrients and the development of soil aggregates (Lian et al., 2019; Ali et al., 2020a; Bu et al., 2020), consequently, influencing soil fertility and plant growth (Tardy et al., 2015). Microorganisms have a distinct role in the decomposition and degradation of organic matter through extracellular enzyme and degrade the macromolecule to monomers to be utilized by the plant (Bending et al., 2004; Harris, 2009). Furthermore, the degradation of plant residues by microorganisms also leads to soil nutrient turnover and circulation, such as carbon (C) and nitrogen (N) cycling and soil aggregate formation (Brennan and Acosta-Martinez, 2017). Several studies reported that the soil pH, organic matter, and other soil properties influenced the soil community composition (Eldridge et al., 2015; Creamer et al., 2016; Pan et al., 2020). Previously, it is well reported that biochar application in combination with N fertilizer improves soil physiochemical properties and crop production (Ali et al., 2020a,b, 2021; Ullah et al., 2021a,b). However, the information on biochar in combination with synthetic N fertilizer on soil microbial and fungal community structure and composition under paddy field condition is not well reported.

Biochar is a carbon-rich, stable product that is produced by the burning of organic material (biomass) of agricultural and forestry wastes, animal bones, algae, and animal manures *via* a controlled process called pyrolysis (Lehmann and Joseph, 2015). Biochar has aromatic and heterocyclic C compounds in its chemical structure, making it resistant to microbial degradation (Ameloot et al., 2013; Anyika et al., 2015). As a soil amendment application, biochar has unique physical and chemical features. Biochar can increase crop growth, yield, and quality by improving soil chemical properties, boosting soil microbial biomass, and enhancing microbial growth and reproduction (Agegnehu et al., 2017; Yu et al., 2019). The addition of biochar to soil decreased soil compactness and affected soil water holding capacity and microbial growth (Liu et al., 2017; Li et al., 2018). Soil microbial populations, community structure, and physiological activity can be affected by biochar application to soil due to their sensitivity of soil microbes (Dempster et al., 2012; Dai et al., 2016). In addition, previous studies reported that application of biochar either increased or decreased the activities of the enzymes, which is related to the transformation of N, C, and P in soil (Bailey et al., 2011; Gomez et al., 2014). Furthermore, it is observed that increasing biochar rate proportionally increased soil microbial abundance (Gomez et al., 2014), whereas, Ameloot et al. (2014) reported the contrast results that 49 t biochar per hectare introverted microbial activity and condensed both extractable phospholipid fatty acid (PLFA) concentration and fungal abundance.

Biochar combined with N fertilizers not only significantly mitigated the problems of excessive fertilizer use such as environmental pollution but also improves soil microorganism abundance and soil enzymes (Ali et al., 2020a). This research is based on continuous long-term research in paddy fields under dual cropping systems, with the following research objectives: (i) characterize the influence of biochar and N application on rice grain yield, soil biochemical properties, and microbial community composition and function; (ii) examine the effect of fertilization on the relationships between microbial community and soil environmental factors; and (iii) measure the contributions of different fertilization regimes, soil biochemical traits, and microorganisms to enhance in rice production. The primary goal of this research is to provide a theoretical framework for sustainable agriculture practices to improve rice production with the use of biochar under lower chemical fertilizers.

## Materials and Methods

### Site Description

The study was conducted at the research farm of Guangxi University, China (22°49′12″ N, 108°19′11″ E; 75 m), in 2019–2020. The climate is classified as subtropical monsoon, and the mean temperature and mean precipitation values of both years are shown in Table 1 (local weather station). The soil (0–20 cm) is graded as Ultisols and is slightly acidic (pH 5.94), soil organic carbon (SOC) 15.10 g kg^–1^, soil organic matter 25.8 g kg^–1^, total N (TN) 1.35 g kg^–1^, available N (AN) 134.7 mg kg^–1^, available phosphorous (23.1 mg kg^–1^), and available potassium (AK 233.6 mg kg^–1^, with 1.36 g cm^–3^ soil bulk density (BD).

**TABLE 1:** Mean temperature and mean precipitation during both year.

Year	2019		2020	
	Mean	Mean	Mean	Mean
Months	Temp (°C)	Precipitation (mm)	Temp (°C)	Precipitation (mm)
Jan	17	98	16	80
Feb	20	102	19	94
Mar	21	72	22	73
Apr	26	92	25	75
May	30	176	29	160
Jun	31	211	30	210
Jul	32	231	34	215
Aug	30	151	31	128
Sep	29	115	28	85
Oct	28	98	28	75
Nov	24	110	23	81
Dec	19	107	17	91

### Biochar Production

Cassava straw was used in kilns with the temperature ranging 300–500°C by following the method previously documented by Mia et al. (2015). The properties of biochar were C (674.00 g kg^–1^), H (3.81 g kg^–1^), P (46.33 g kg^–1^), N (5.43 g kg^–1^), K (48.33 g kg^–1^), S (2.39 g kg^–1^), specific area (2.46 m^2^ g^–1^), and pore diameter (3.37 nm) with C:N ratio (124.12.) and are presented in our previous study (Ali et al., 2020a).

### Experimental Design

The field experiment was conducted in a randomized complete block (RCB) design having three replications and a plot size of 3.9 by 6 m (23 m^–2^) during 2019 and 2020. The experiment consisted of four biochar rates (0, 10, 20, and 30 ton ha^–1^) and two N levels (135 and 180 kg ha^–1^). Biochar amendment was applied once in 2019, whereas N application was applied in both years. The treatments combinations were as follows: T1 = 0 t B + N135 kg ha^–1^, T2 = 0 t B + N180 kg ha^–1^, T3 = 10 t B + N135 kg ha^–1^, T4 = 20 t B + N135 kg ha^–1^, T5 = 30 t B + N135 kg ha^–1^, T6 = 10 t B + N180 kg ha^–1^, T7 = 20 t B + N180 kg ha^–1^, and T8 = 30 t B + N180 kg ha^–1^. The cultivar “Zhenguiai” of noodle rice was utilized as a test crop. Plastic trays were used for the nursery and uniform seedlings were transplanted as two seedlings per hill and 13 rows per plot after 25 days. The locally recommended doses of phosphorus and potassium were applied at the rate of 75 and 150 kg ha^–1^, respectively. The biochar was introduced to the field 25 days before the transplantation of seedlings. At the panicle initiation stage, when plant growth is at its highest, soil samples were collected near the rhizosphere. The soil samples were transported to the lab in an icebox and stored at −80°C for later use.

### Soil Chemical Traits and Microbial Biomass

Soil samples were taken by a core sampler at depth 0–20 cm after the late-season rice harvest in 2019–2020. Soil sampling was done at different locations within each plot and combined to make a composite sample. The composite samples were divided into two parts, with one part frozen at −80°C for later DNA extraction and microbial biomass C and N measurement, and the second half was air-dried and utilized to determine soil chemical characteristics.

SOC was measured by the K_2_Cr_2_O_7_-H_2_SO_4_ oxidation process followed by titration (Wang et al., 2014). To determine soil TN, a subsample of 200 mg was treated using the salicylic acid–sulfuric acid–hydrogen peroxide method previously described by Ohyama et al. (1991), and TN was determined using the micro-Kjeldahl technique according to Jackson (1956). In addition, soil pH and available N, P, and K were assessed by the methods of Lu et al. (2015). The fumigation extraction method was used to measure microbial biomass carbon (MBC) as defined by Brookes et al. (1985) and microbial biomass nitrogen (MBN) according to the method of Vance et al. (1987).

### DNA Extraction and Sequencing

DNA samples were extracted using the Fast DNA™ spin kit for soil (MP Biomedicals, US) following the manufacturer’s instructions. The DNA concentration was measured using NanoDrop 2000 (Thermo Fisher Scientific, Wilmington, DE, United States), and the quality of PCR products was detected by 2% agarose gel electrophoresis. The V3–V4 region of the 16S rRNA gene was amplified with primer pairs 515F (GTGCCAGCMGCCGCGG) and 907R (CCGTCAATTCMTTTRAGTTT). The primer pair Internal transcribed spacer (ITS) 1F (CTTGGTCA- TTTAGAGGAAGTAA) and ITS 2R (GCTGCGTTC- TTCATCGATGC) was used to amplify the ITS 1 region of fungi. The PCR and sequencing processes were performed by Majorbio Bio-Pharm Technology Co., Ltd. (Shanghai, China) using the Illumina MiSeq PE300 platform. The data were analyzed on the free Majorbio Cloud Platform.^1^

### Processing of Illumina Sequencing Data

The paired reads were spliced using FLASH (version 1.2.3) software to merge the sequences before assembling a gene segment (Magoc and Salzberg, 2011). Chimeric sequences were identified and removed with a *de novo* method using USEARCH (version 8.1.1861) (Edgar, 2010). After the removal of the chimera, high-quality bacterial sequences were collected for subsequent analysis.

Effective bacterial sequences were separately subsampled for each sample for the subsequent statistical analysis. After subsampling, the data were processed using a modified SOP pipeline on the basis of USEARCH and the software package QIIME (Quantitative Insights Into Microbial Ecology v1.8.0) (Tian et al., 2015). Briefly, the selected sequences were clustered to operational taxonomic units (OTU) using a two-stage clustering algorithm with USEARCH (version 8.1.1861) at 97% sequence identity (Edgar, 2010). Representative sequences in each OTU were aligned to the SILVA reference alignment (Yilmaz et al., 2014). Taxonomy was assigned to each representative sequence using RDP with a minimum confidence of 85%.

### Alpha and Beta Diversity Analysis

An OTU-based analysis method was used to evaluate the bacterial diversities in each sample from each plant (alpha diversity). To estimate the diversity index and species richness (alpha diversity) among the genotypes for each sample, OTU richness and Chao1, Simpson, and Shannon indices were calculated using QIIME software (v1.8.0), concerning a sequencing depth of 3%. Statistical analysis was performed using ANOVA with *p*-values to determine the significant differences in the diversity indices or species richness among the plant rhizosphere soil samples. The rarefaction curve and rank abundance curves were calculated at a 97% level of similarity of the OTUs.

Beta diversity analysis was used among all the samples for the similarity index determination of the community structure. At the OTU level of genotypes, beta diversity was calculated using weighted UniFrac distances and was visualized through PCoA (principal coordinate analysis). The weighted UniFrac distance matrices were clustered and evaluated by QIIME software (v1.8.0) and showed phylogenetic relationships among various communities and their abundance in the respective samples.

### Rice Grain Yield

At maturity, the rice plants were harvested from the whole plot and rice grain yields was weighed. The dry weight of the rice grain was determined assuming adjusted 14% moisture content in rice grains.

### Statistical Analysis

Statistics 8.1 analytical software was used to determine the analysis of variance among the treatments for each variable. Alpha diversity of bacteria and fungi including Simpson, Shannon, Chao1, and ACE indices was calculated using QIIME software (v1.8.0). Rarefaction curves of the species richness were plotted against the number of sequences, and the analysis of the dominant phyla was done using the Microbiome Analyst (Dhariwal et al., 2017). Redundancy analysis (RDA) was performed using the software package CANOCO5 (Microcomputer Power, Ithaca, United States) to measure soil properties and soil microbial diversity relationship. R (3.2) software was used to conduct correlation analysis among treatments for soil microbial abundance, soil properties, and grain yield. SmartPls3 software was used to analyze consistence multi-group analysis (MGAc) among the treatments for all attributes.

## Results

### Soil Chemical Traits and Microbial Biomass

Co-application of biochar and mineral N significantly improved the soil pH, SOC, TN, MBC, and MBN in 0–20 cm soil depth, compared with sole chemical N fertilizer application (Table 2). In all measured traits, the effect was highest under high biochar amendment input with no significant differences between 20 and 30 t ha^–1^ of biochar application. Across the years, the treatments exhibited the same behavior. Compared to sole N-treated plots (T1 and T2), higher biochar applied treatments (T4 and T8) improved soil pH, TN, SOC, SMBN, and SMBC by 15, 38, 26.17, 94, and 129% respectively, during 2019. Similarly, in 2020, soil pH, TN, SOC, SMBN, and SMBC were increased by 16, 44, and 32% in T4, respectively, as compared to T1. Whereas soil BD was decreased by 7.5 and 9% in non-biochar treatments (T1 and T2) as compared to higher biochar applied treatment (T4). SMBN was averagely increased in T4, T7, and T8 by 50, 80, and 95%, respectively, during both years as compared to T1. Similarly, SMBC was averagely enhanced by 134, 126, and 128% in T4, T7, and T8, respectively, as compared to T1 during both years.

**TABLE 2:** Response of soil properties to different biochar and nitrogen fertilizer treatments.

Years	Treatments	pH (water)	TN (g kg^–1^)	SOC (g kg^–1^)	BD (g kg^–1^)	SMBN (mg kg^–1^)	SMBC (mg kg^–1^)
2019	T1	6.12c	1.40c	13.23c	1.35a	22.33d	174.9c
	T2	6.41b	1.50bc	12.95c	1.35a	26.66c	294.1b
	T3	6.49b	1.57b	15.14b	1.30b	30.66c	300.7b
	T4	7.08a	1.60b	16.06ab	1.27bc	33.66c	384a
	T5	6.23c	1.61b	16.55ab	1.26c	41b	410a
	T6	6.46b	1.87a	16.46ab	1.28bc	29.33c	314.0b
	T7	7.09a	1.95a	16.77ab	1.26c	40.33a	396.6a
	T8	7.12a	1.96a	17.11a	1.24d	43.66a	400.3a
	Lsd	0.32	0.11	1.64	0.05	8.11	42.4
	T1	6.00c	1.37c	12.54c	1.33a	24.66d	167c
2020	T2	6.37bc	1.47c	11.73c	1.30b	29c	187.6c
	T3	6.44ab	1.60b	14.99b	1.28bc	33.66b	281.6b
	T4	7.09a	1.63b	16.25ab	1.25bc	36.66b	357a
	T5	6.08b	1.64b	16.36ab	1.23bc	44.33a	367a
	T6	6.36a	1.89a	16.33ab	1.24bc	32.33b	259b
	T7	6.99ab	1.98a	16.64a	1.22c	43a	340.6a
	T8	7.02a	1.99a	16.65a	1.22c	47.33a	341.6a
	Lsd	0.26	0.12	1.52	0.07	7.49	38.59

*TN, total nitrogen; SOC, soil organic carbon; BD, bulk density; MBC, microbial biomass carbon; and MBN, microbial biomass nitrogen. Within a column, values preceded by the same letters are not significantly different at p ≤ 0.05. Note: T1 = 0 t B + N135 kg ha^1^, T2 = 0 t B + N180 kg ha^1^, T3 = 10 t B + N135 kg ha^–1^, T4 = 20 t B + N135 kg ha^–1^, T5 = 30 t B + N135 kg ha^1^, T6 = 10 t B + N180 kg ha^1^, T7 = 20 t B + N180 kg ha^–1^, and T8 = 30 t B + N180 kg ha^–1^.*

### Sequencing Quality Control and Summary

**FIGURE 1:**
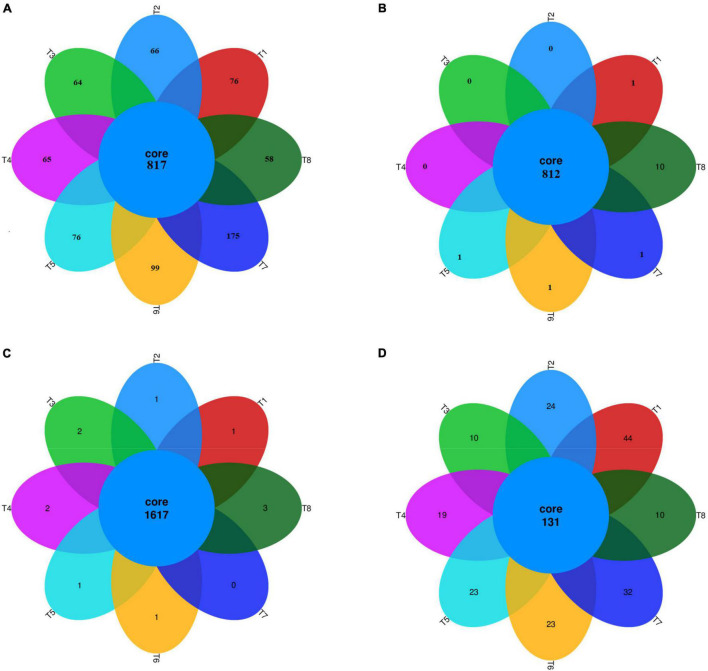
Venn diagram. A Venn diagram representing unique and shred OTU’s of bacteria and fungi during 2019 **(A,C)** and 2020 **(B,D)**, respectively, among the different treatments. T1 = 0 t B + N135 kg ha^1^, T2 = 0 t B + N180 kg ha^1^, T3 = 10 t B + N135 kg ha^–1^, T4 = 20 t B + N135 kg ha^–1^, T5 = 30 t B + N135 kg ha^1^, T6 = 10 t B + N180 kg ha^1^, T7 = 20 t B + N180 kg ha^–1^, and T8 = 30 t B + N180 kg ha^–1^.

After screening, pre-clustering, and chimera removal, a total of 1,318,797 reads of high-quality bacterial 16S rRNA from the V3–V4 region were obtained, with an average of 54,949 reads per sample, having 32 phyla, 75 classes, 107 orders, 168 families, and 250 genera during 2019. Whereas, during 2020, a total of 763,986 reads were amplified with an average reads of 31,832 per sample, having 25 phyla, 66 classes, 100 orders, 170 families, and 253 genera during 2020. The unique numbers of OTUs were 76, 66, 64, 65, 76, 99, 175, and 58 in T1, T2, T3, T4, T5, T6, T7, and T8, respectively (Figure 1A). In 2020, the unique numbers of OTUs were 1, 1, 1, 1 and 10 in T1, T5, T6, T7, and T8, respective (Figure 1B).

A total of 756,790 reads were amplified for the fungal population, with an average reads per sample 31,532, having 10 phyla, 22 classes, 50 orders, 74 families, 94 genera, and 102 species, during 2019. The unique numbers of OTUs were 1, 1, 2, 2, 1, 1, 0, and 3 in T1, T2, T3, T4, T5, T6, T7, and T8, respectively (Figure 1C). Similarly, during 2020, a total of 404,210 reads were amplified with an average reads per sample of 16,842, having 10 phyla, 20 classes, 47 orders, 69 families, 84 genera, and 94 species. The unique numbers of OTUs were 44, 24, 10, 19, 23, 23, 32, and 10 in T1, T2, T3, T4, T5, T6, T7, and T8, respectively (Figure 1D). The results showed that the fungal OTU numbers were significantly decreased in biochar applied treatments as compared to other treatments (Supplementary Files-Presentation 1- and Table 1).

### Composition and Community Structure of the Rice Rhizosphere Microbiomes Under Different Biochar and Nitrogen Application

**FIGURE 2:**
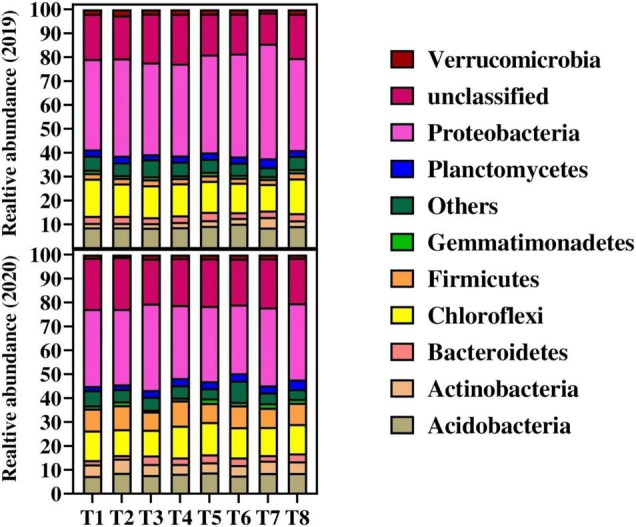
Changes in relative abundance on bacterial species on phylum level in response to different treatments. Distinct colored columns represent different species, and the length of the columns represents the species’ proportion. For treatment combination details (see Table 1).

The bacterial relative abundance of major bacterial phylum in each treatment is shown in Figure 2. The dominant bacterial phylum across all the treatments was Proteobacteria, Acidobacteria, Actinobacteria, Bacteroidetes, Gemmatimonadetes, Planctomycetes, Verrucomicrobia, and Chloroflexi among the others. The results showed that Proteobacteria were more (> 5%) abundant in all the treatments. Higher relative abundance of Proteobacteria was found in T7 and T3 during 2019 and 2020, respectively, compared to other treatments. The second most abundant bacteria were Chloroflexi and were not significantly influenced by biochar and N fertilizer. Among the treatments, Actinobacteria, Proteobacteria, and Verrucomicrobia were higher in relative abundance ranging from 4.3, 47.98, and 1.3% in T7 as compared to control treatment (T1) in 2019. Similarly, compared to control (T1), Acidobacteria and unclassified bacteria were observed higher in relative abundance ranging from 17.56 and 10.23% in T6 and T4, respectively, during 2019 as compared to the control treatments. Furthermore, the relative abundance of Bacteroidetes, Gemmatimonadetes, and Planctomycetes were higher ranging from 20.64, 7.85, and 6.96%, respectively, in T5 over control treatment (T1) in 2019. Chloroflexi and Firmicutes resulted in higher relative abundance by 9.85 and 9.81%, respectively, in T8 and then control treatment (T1) during 2019. Whereas, during 2020, Acidobacteria, Actinobacteria, Bacteroidetes, Gemmatimonadetes, Planctomycetes, and Verrucomicrobia resulted higher in biochar plots by 12.4, 7.15, 99.41, 62.7, 74.92, and 20.6%, respectively, as compared to control (T1) (Figure 2). Chloroflexi, Firmicutes, Proteobacteria, and unclassified bacteria were not significantly influenced by biochar and N application during 2020, where between the years, Chloroflexi, Firmicutes, and Proteobacteria were decreased in 2020 as compared to 2019.

**FIGURE 3:**
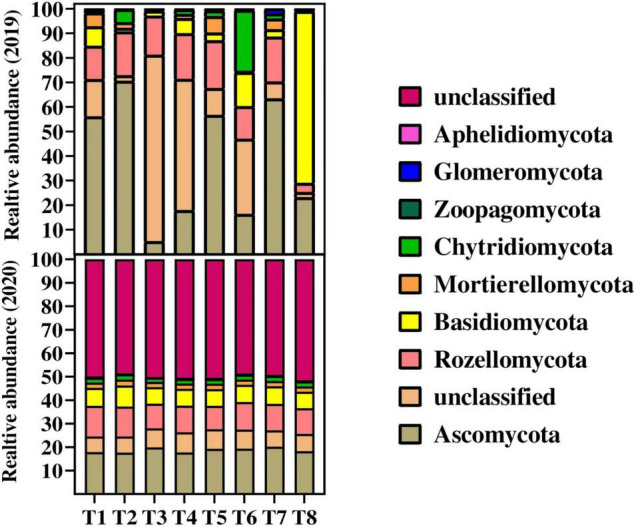
Changes in relative abundance on fungal species on phylum level in to different treatments. Distinct colored columns represent different species, and the length of the columns represents the species’ proportion. For treatment combination details (see Table 1).

The relative abundance of fungal species at phylum level among all the treatments during 2019 and 2020 is shown in Figure 3. The results showed that the relative abundance of major fungal phylum was decreased with the increase in biochar application in the second year. During both years, the Ascomycota fungi were the most abundant fungi followed by Rozellomycota, Basidiomycota, Mortierellomycota, Chytridiomycota, Zoopagomycota, and Glomeromycota across the treatments. The overall results showed that the relative abundance of phylum Ascomycota, Rozellomycota, and Basidiomycota fungi were significantly decreased in 2020 as compared to 2019. Among the treatments, the results showed that the lowest 5 and 17% relative abundance of Ascomycota was recorded in T3 (20 t B ha^–1^ under + 135 kg N ha^–1^) and T4 (30 t B ha^–1^ under + 135 kg N ha^–1^), respectively, as compared to control and other treatments during 2019. Similarly, a Chytridiomycota fungus was recorded less of relative abundance ranging from 0.34 and 1.91% in T3 and T4, respectively, as compared the rest treatments during 2019. Whereas during 2020, unclassified fungi were higher in T3, T4, T5, and T8 with the abundance of 50.08, 50.46, 50.44, and 51.46%, respectively. The lowest values of 48, 49.92, and 48.56% were recorded in T1, T2, and T6, respectively, for unclassified fungal abundance. Furthermore, the higher fungal species at phylum level followed the order Ascomycota > unclassified fungi > Rozellomycota > Basidiomycota > Mortierellomycota > Chytridiomycot Zoopagomycot > Glomeromycota > Aphelidiomycota across the samples.

**FIGURE 4:**
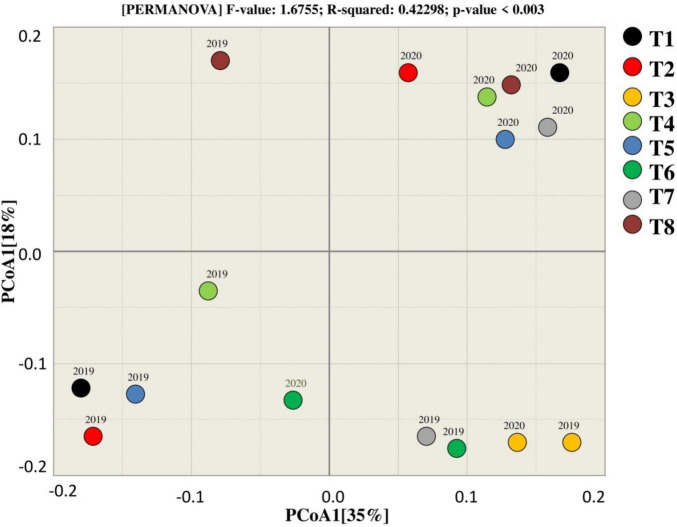
Beta diversity analysis for estimating similarity and dissimilarity among the treatments for the fungal community.

The bacterial diversity at genus level influenced by different biochar and N fertilizer during 2019 is represented in Supplementary File 1. Presentation 1 and Figure 1. A total of 250 classified species were found among all soil samples. *Thiobacter* was the most abundant bacteria at genus level after unclassified bacteria, followed by *Sphingopyxis*, *GP6*, *Geobacter Povalibacter*, *Sphingosinicella*, *Gemmatimonas*, *Aminicenantes genera incertae sedis*, *Gp18*, *Bellilinea*, and *Kofleria.* Among the treatments, T7 resulted in higher percent of *Sphingopyxis*, whereas T3 resulted in maximum percent of *Thiobacter* bacteria. Whereas, during 2020, a total number of classified bacteria species at genus level were 179, and the most abundant bacteria after unclassified were *Novosphingobium* followed by *Fictibacillus*, *Bacillus*, *Sphingomonas*, *Gp6*, *Clostridiumsensu stricto*, *Gemmatimonas*, *Aminicenantes genera incertae sedis*, *Thiobacillus*, *Spartobacteria genera incertae sedis*, *Gp16*, *Reyranella*, *Geobacter*, *Gp17*, *Streptomyces*, *Novosphingobium*, *Fictibacillus*, *Methanothrix*, and *Lysinibacillus* (Supplementary File 1. Presentation 1 and Figure 2). Among the treatments, T7 and T5 resulted in higher percent of *Bacillus* bacteria. Furthermore, *Sphingomonas* was higher in T3 as compared to other treatments. The overall results showed that the classified fungi were decreased in 2020 as compared to 2019. Similarly, for fungal genus abundance, the top 10 fungi at genus level were *Gaeumannomyces*, *Myrothecium*, *Zopfiella*, unclassified *Pleosporales*, *Mortierella*, *Pyrenochaetopsis*, *Aspergillus*, unclassified *Mortierellales*, *Fusarium*, and *Cladosporium* among 49 species during 2019 (Supplementary File 1. Presentation 1 and Figure 3). Whereas in 2020, the top 10 most abundant fungal species at genus level were *Echria*, *Panaeolus*, *Westerdykella*, *Tomentellopsis*, *Calocybella*, *Kohlmeyeriopsis*, *Mortierella*, *Zopfiella*, *Gaeumannomyces*, and *Schizothecium* among 37 species (Supplementary File 1. Presentation 1 and Figure 4).

### Alpha Diversity

**FIGURE 5:**
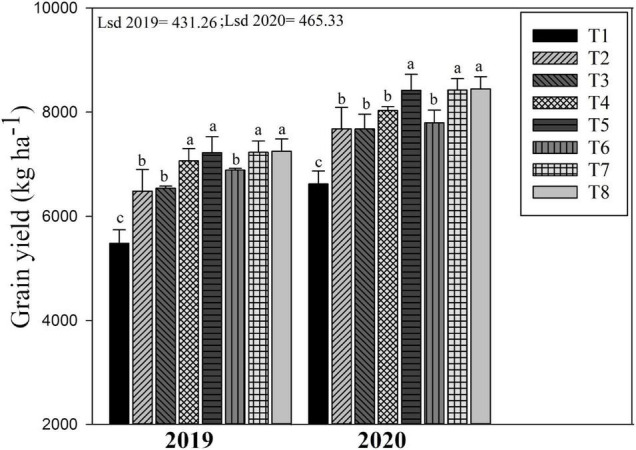
Changes in grain yield of rice as influenced by different biochar rates in combination with different N fertilizers. The mean comparison was made using the least significant difference (LSD) test for treatments means based on the LSD test at 5%. Different letters on bars are not significantly different at *p* < 0.05. For treatment combination details (see Table 1).

**FIGURE 6:**
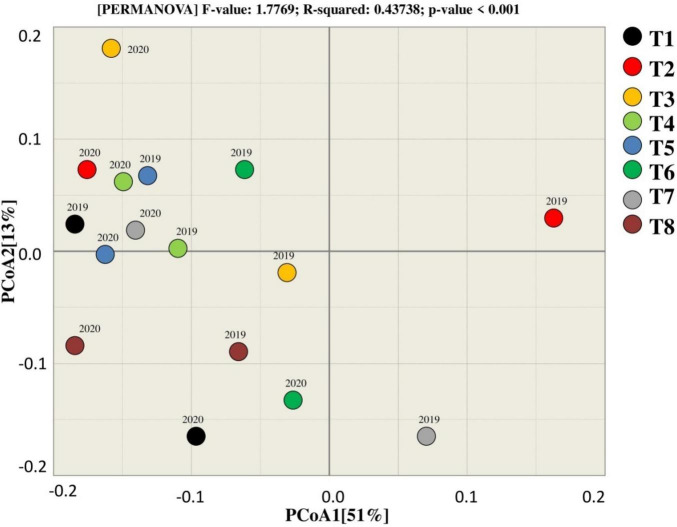
Beta diversity analysis for estimating similarity and dissimilarity among the treatments for bacterial.

**FIGURE 7:**
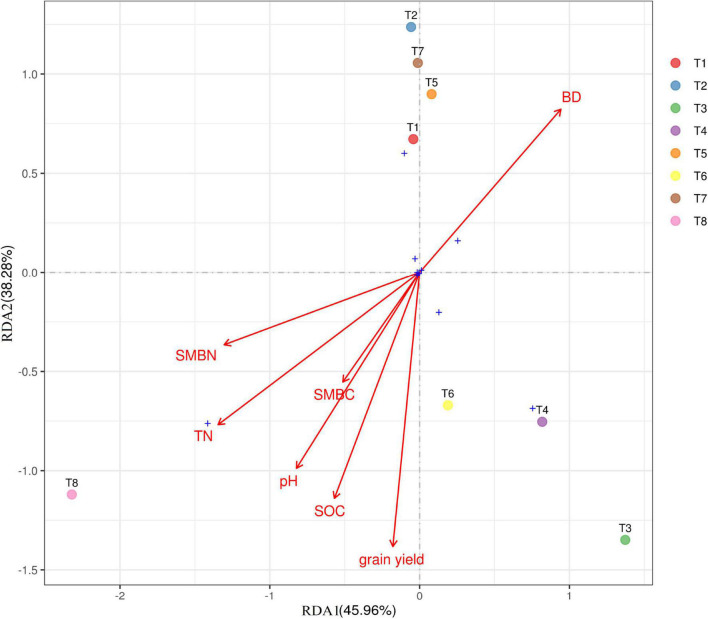
Ordination plot of results from redundancy analysis to identify relationship between soil properties, grain yield (GY), and dominant bacterial phylum among the treatments. SMBN, soil microbial nitrogen; SMC, soil microbial carbon; TN, total nitrogen; SOC, soil organic carbon. For treatment combination details (see Table 1).

**FIGURE 8:**
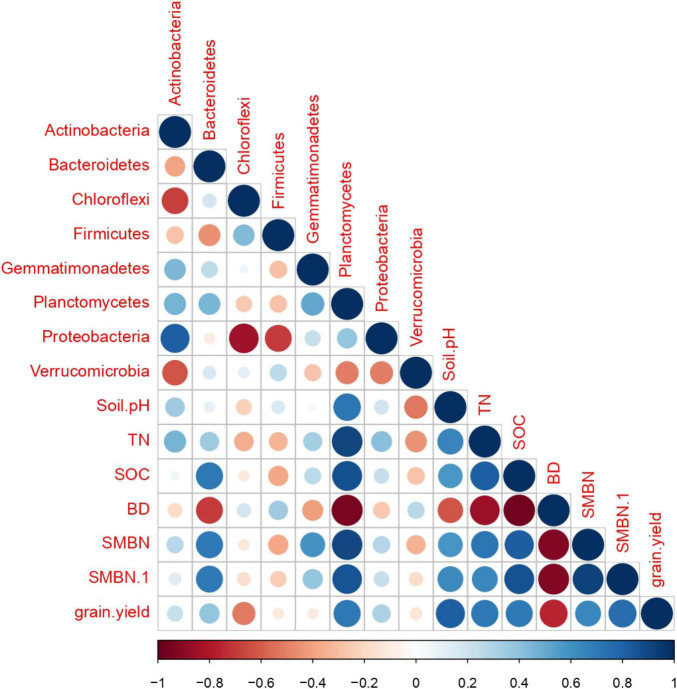
Correlation analysis among treatments for soil microbial abundance, soil properties, and grain yield.

**FIGURE 9:**
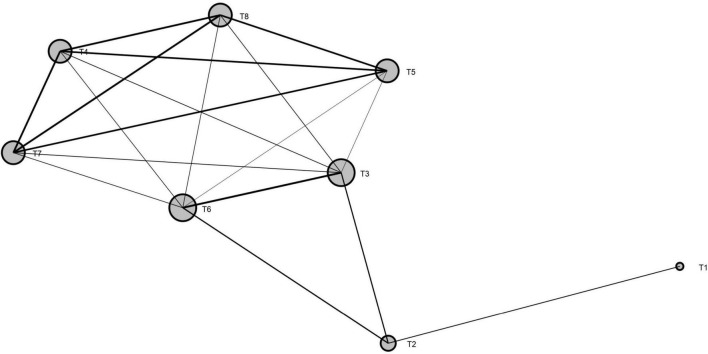
Network plot (MGAc analysis) among the treatments for soil microbial abundance, soil properties, and grain yield. Size of the ball represents the variation among the treatments, and lines represent the interaction among the treatments. Note: T1 = 0 t B + N135 kg ha^1^, T2 = 0 t B + N180 kg ha^1^, T3 = 10 t B + N135 kg ha^–1^, T4 = 20 t B + N135 kg ha^–1^, T5 = 30 t B + N135 kg ha^1^, T6 = 10 t B + N180 kg ha^1^, T7 = 20 t B + N180 kg ha^–1^, and T8 = 30 t B + N180 kg ha^–1^.

To assess the diversity among the treatments, alpha diversity indices were calculated for each samples (Supplementary File 1. Presentation 1, Table 2 and Figures 5–6). The rarefaction curve illustrated enough richness of observed OUTs and sequencing depth to examine microbial alpha diversity (Supplementary File 1. Presentation 1 and Figures 7–10). According to the results Chao1, ACE, Shannon, and Simpson indices were decreased in 2020 as compared to 2019. In 2019, the control treatment showed slightly higher OTU richness (Chao1 = 15,459.03) as compared other treatments. The lowest OTU richness (Chao1 = 13,678.0) observed in T7 as compared to the rest of treatments (Supplementary File 1. Presentation 1-Table 2 and Figures 6A–D). Whereas during 2020, the OTU richness was higher in T7 (Chao1 = 1,467.1), and the lowest OTU richness (Chao1 = 1,425.67) in T2. The higher ACE index of 2,299.07 and 1,459.30 was recorded in T2 in 2019 and 2020, respectively. The lowest ACE index of 2,205.90 and 1,417.06 was recorded in T6 and T7 during 2019 and 2020, respectively. Shannon and Simpson indices were recorded lowest in T6 (6.88) and T7 (0.995) as compared to the rest of treatments in 2019, whereas, in 2020, the Shannon and Simpson indices increased with biochar application as compared to control treatments.

In addition, the results of alpha diversity for fungi showed the opposite trend as compared to bacterial. The diversity indices among fungal samples were higher in 2019 and lower in 2020. Among the treatments, T4 resulted in a higher OTU richness (Choa1 = 190.36) in 2019, whereas T5 resulted in higher OTU richness (Chao1 = 956.51) in 2020. The lowest OTU richness for fungal taxonomic feature level was recorded in T6 (Chao1 = 151.05) and T2 (Chao1 = 920.3) during 2019 and 2020, respectively. Shannon and Simpson indices were not significantly affected by biochar and N treatments as compared to control treatments. However, variation do exist among the samples, for example, the higher Shannon index was recorded in T5 during 2019 and T4 during 2020.

### Beta Diversity

PCoA was used to assess the similarity and dissimilarity for bacterial beta diversity among the treatments. According to the results, most of the samples of corresponding treatments tend to group, indicating that there is a similarity between the treatments as they clustered near to each other except T7 that tends to differ from other treatments in terms of rhizosphere microbial community. Moreover, 51% variation among the treatments was explained by PCoA1, whereas PCoA2 explains 13% of the total variation among the treatments during both years (Figure 6).

Beta diversity analysis (PCoA) of fungal community showed that the treatments tend to the group, presenting that there is a connection among the treatments as they clustered near to each other except T8 that tends to differ from other treatments in terms of rhizosphere during 2019. The results showed that treatments in 2019 and 2020 clustered in different quadrate that indicates that the difference among the treatments of in both years. T3, T6, and T2 were observed together in two different groups, which represents that these treatments were dissimilar for the fungal community in 2019. The variation among the treatments by PCoA1 and PCoA2 was explained by 35 and 18%, respectively, for the fungal community in both years.

### Grain Yield

The biochar and N fertilizer combined application considerably affect the rice yield in both years (Figure 5). Biochar applied treatment including T8, T7, and T5 improved rice yield by 32.4, 31.8, and 31.7% as compared to non-biochar applied treatments (T1) in 2019. Similarly, in 2020, the grain yield of rice was higher in T8, T7, and T5 by 27.2, 23.5, and 27.6%, respectively, as compared to T1. Compared to T2 (no biochar + 180 kg ha^–1^), the treatments T8, T7, and T5 significantly increased grain yield by 10.46, 10.56, and 10.87%, respectively, during both years. The lowest grain yield of rice during both years was recorded in non-biochar–treated plots (T1 and T2).

### Relationship Between Bacterial Community Composition and Soil Properties

RDA was performed to determine the strength of the association between the soil pH, SOC, TN, BD, SMBN, and SMBC contents and the diversity of the soil bacterial composition. Figure 7 shows the relationship between bacterial communities (at phylum level) and the soil properties for the different treatments. Soil pH, TN, SOC, SMBN, SMBC, and grain yield occurred in same quadrant, which indicates that biochar has significant effect soil physiochemical properties and grain yield. The eight treatments took place in four different quadrants, showing that the fertilization treatments had a substantial effect on the composition of soil bacterial composition. Biochar applied at higher rate (T8) showed significant correlation with soil properties.

Figure 8 shows the Pearson correlation heatmap among the most abundant bacteria’s, soil properties, and grain yield. Soil properties including soil pH (*R* = 0.82), TN (0.72), SOC (0.71), SMBC (0.64), and SMBN (0.76) were strongly positively correlated with grain yield. However, the relationship between soil BD (−0.75) and grain yield was strongly negatively correlated. Furthermore, the abundance of soil bacteria including *Acidobacteria* (0.37), Actinobacteria (0.24), Bacteroidetes (0.39), Planctomycetes (0.71), and Proteobacteria (0.32) strongly positively correlated with the grain yield of paddy rice, whereas, the abundance of Chloroflexi, Firmicutes, Gemmatimonadetes, and Verrucomicrobia showed no significant relationship with grain yield of rice. The relationship between the soil biochemical properties and soil bacteria abundance were also positively correlated, except soil BD.

For the measured indicators of all soil bacterial abundance, soil properties, and grain yield, a network plot (MGAc) among the treatments was created to understand the relationship among the treatments using SmartPls3 software (Figure 9). The results showed that, for all measured traits, the treatments without biochar applications (T1 and T2) were significantly dissimilar from the treatments with biochar application, whereas the treatments had biochar rate from 20 to 30 ton ha^–1^ under both N fertilizers (T4, T5, T6, and T8) were resulted in the same outputs for all traits across the years.

## Discussion

Biochar application and the use of N fertilizer are important agricultural management practices in sustainable development. Several reports have concluded that the use of biochar considerably improves soil health and crops yield. However, the effect of long-term biochar application in combination with chemical N fertilizer on paddy field soil properties and microbial community composition during 2 years is still unclear. This research explored the effect of biochar in combination with N fertilizer on paddy field soil properties, microbial functions, and bacterial community composition. The soil bacterial community is the essential component of soil ecology, responsible for enhancing soil health, and plants production (Luo et al., 2020).

### Soil Properties

In the present study, biochar amendment in combination with N fertilizer significantly improved soil biochemical indicators (i.e., MBC, MBN, pH, SOC, and TN) compared with sole N fertilization (Table 2), whereas soil DB was decreased in biochar-treated soil as compared to sole N-applied treatments. The possible explanation for this increment might due to the biochar’s higher porosity, higher surface area, and its large number of microspores (Ali et al., 2020a,b, 2021). Our results were in line with our previous study (Ali et al., 2021), showing that the biochar addition in combination with urea considerably improved the pH by 14%. Numerous studies have shown that soil pH can increase when biochar is applied, particularly in acidic soils, which can ameliorate the nutrient supply to plants (Ali et al., 2020a,b; Ullah et al., 2021a,b). Similarly, SMBN and SMBC were increased might due to the alkaline nature of biochar. Biochar amendment addition to acidic soils can improved the microbial activities and increases the microbe populations as documented in our previous study (Ali et al., 2021). The other possible explanation might be due to the inhibition of denitrification inhibitors, which are the major regulators of nitrification. Our findings are supported by the previous results of Zhou et al. (2017), who documented that SMBC and SMBN can be increased by 26 and 21% in biochar-treated soil as compared to control. Liu et al. (2016) also reported that biochar application improved soil MBN and MBC as compared to non-biochar applied treatments. However, in contrast to our results, Castaldi et al. (2011), Zavalloni et al. (2011), and Dempster et al. (2012) have documented that biochar has no significant effects on soil MBC.

### Impact of Biochar Amendment in Combination With Nitrogen Fertilizer on the Abundances of Soil Bacteria and Fungi

The microbial population’s diversity and richness are regarded critical for soil integrity, functionality, and sustainability, yet they are commonly diminished by current farming practices (Zhao et al., 2014; Khan et al., 2021). In the present study, the different biochar rates in combination with N fertilizer significantly affected the soil bacterial and fungal abundance (Table 2). Compared to control (T1), biochar application at the rate of 20–30 t ha^–1^ significantly increased soil bacterial abundance. The possible reason for these increments due to the increase in soil pH in biochar applied treatments. Several studies reported that soil physical and chemical properties indirectly affect soil microbial abundance (Lehmann et al., 2011; Cole et al., 2019). For example, soil pH is the most important factor for the change in bacterial abundance of soil (Yao et al., 2017; Liu et al., 2019; Zheng et al., 2019; Sun et al., 2020). Thus, biochar in combination with N fertilizer improved soil pH in our experiment, which consequently improved soil bacterial abundance. A similar study was reported by Yao et al. (2017) in that the addition of a higher rate of biochar increases bacterial abundance. Likewise, Chen et al. (2015) also reported that 40 tons of biochar ha^–1^ significantly increased bacterial 16S rRNA gene copy numbers by 35–62%. However, Luo et al. (2017) and Gao et al. (2021) reported that that biochar fertilizer in alkaline soil did not affect soil pH or bacterial abundance. Thus, our results suggested that an appropriate rate of biochar application in combination with N fertilizer improved soil bacterial abundance in the paddy field.

In this study, fungal abundance was decreased in the biochar-treated soil as compared to control. This increase might be due to an increase in soil pH as compared to control in biochar-treated soil. Yao et al. (2017) reported a similar finding that changes in soil fungus might be caused by changes in soil pH and nutrient content due to biochar addition. Because biochar has a higher pH, its effect would be exacerbated in acidic soil, where the changes in soil pH after applying biochar amendment would be more significant (Paz-Ferreiro et al., 2015; Xu et al., 2018). In contrast, Steinbeiss et al. (2009), Jones et al. (2012), Luo et al. (2017), and Gao et al. (2021), reported that biochar amendment could promote fungal growth as compared with the control soil.

### Community Compositions of Soil Bacteria and Fungi Influences by Biochar and Nitrogen Application

Many studies have shown that biochar has a short- or long-term impact on bacterial and fungal community compositions (Yao et al., 2017; Li et al., 2020). However, the effects of biochar on community composition remain unclear. In the present study, the abundance of Proteobacteria, Actinobacteria, and Verrucomicrobia were observed higher in biochar-treated soil as compared to control. In terms of community composition and relative abundance, Proteobacteria accounted for the highest fraction in the soil, which is consistent with the findings (Ji et al., 2016; Yin et al., 2021). For a possible explanation, Proteobacteria are a eutrophic bacterium (Fierer et al., 2007), and, previously, it is well documented that biochar amendment improves soil properties (Ali et al., 2020a,b, 2021), leading to an increase in the Proteobacteria population.

The relative abundance of Acidobacteria, Chloroflexi, Actinobacteria, Bacteroidetes, Gemmatimonadetes, Planctomycetes, Verrucomicrobia, and Chloroflexi was higher in the first year of the experiment. However, in 2020, the frequency of these microbes has reduced. Most Acidobacteria are acidophilic bacteria, and their relative abundance is negatively correlated with soil pH. In our study, biochar increased soil pH as compared to control, which consequently decreased Acidobacteria. Similar reducing results of Acidobacteria abundance in higher soil pH (8.50 and 7.87) compared to low soil pH (5.35) were reported by Yin et al. (2021).

The relative abundance of Actinobacteria was increased in biochar-treated soil as compared to control. As gram-positive bacteria, Actinobacteria play a significant role in organic matter turnover, including cellulose and chitin decomposition (Ali et al., 2019). Bacteroidetes abundance was improved in the second year as compared to first year and was higher in biochar applied treatments as compared to control. The application of biochar to the soil as a carbon source promotes an increase in the relative abundance of Bacteroidetes in the soil, because Bacteroidetes bacteria are strongly correlated with the conversion of organic materials such as DNA, proteins, and lipids (Cottrell and Kirchman, 2000).

Biochar in combination with N fertilizer significantly affected the abundance of fungal community structure (Figure 3). In the present study, the dominant fungal phyla across the treatments were Ascomycota, Rozellomycota, Basidiomycota, Mortierellomycota, Chytridiomycota, Zoopagomycota, and Glomeromycota during both years. Compared to control, biochar application in combination with N fertilizer significantly decreases the relative abundance of Ascomycota, Rozellomycota, and Basidiomycota phyla, whereas, between the years, these phyla were more decreased in 2020 over 2019. The possible explanation for these condense in fungal phyla might due to changes in soil chemical and physicochemical properties, especially soil pH. Furthermore, (Heitkötter and Marschner, 2015), Ullah et al. (2020) and Ali et al. (2020a,b), reported that biochar changes in soil physicochemical properties alter soil enzyme activities, which consequently resulted in the abundance and composition of soil fungi abundance and composition. Compared to 2019, reduction in Ascomycota during 2020 might be attributed to that biochar as a slow-release fertilizer can frequently take up to a year to see results. Further explanation for these changes might be that biochar as a microbial C source; the Dissolved organic carbon (DOC) probably promotes saprotroph growth and enhances their competitive capacity, leading to an overall decrease in diversity and a decline in fungal pathogens (Dai et al., 2018). Similar results to our finding were reported by Yin et al. (2021) that biochar decreased the relative abundance of Ascomycota, Basidiomycota, and Mortierellomycota. Another study reported that these fungi decompose organic matter, symbiotic fungi, parasitic or pathogenic fungi, and even other fungi (Hang et al., 2020; Liu et al., 2020). Furthermore, Chen et al. (2013) and Hale et al. (2014) documented that because of biochar porous nature protects soil from a variety of biological rivals. In addition, our study also reported that Mortierellomycota phyla were decreased in biochar-treated soil during both years. The possible reason for this change due to biochar provides carbon supply to the soil, and it is hypothesized that dominant fungal genera in soil are competing for carbon source, leading to a decrease in Mortierellomycota. Same results to our findings were observed by Yin et al. (2021) that Mortierella abundance was observed lesser in biochar-treated soil as compared to control.

### Grain Yield

Co-incorporation of biochar and synthetic N fertilizer significantly increased rice grain yield compared to non-biochar applied plots (Figure 5). The enhancements in rice yield could be attributed to enhancement in soil pH, TN, SOC, MBC, and MBN (Table 2), which ultimately enhanced rice growth and biomass accretion by providing enough nutrients during the growing period. The results of the Pearson correlation heatmap confirmed that that the variations in soil nutrients were the factors that elucidated the greatest proportion of the difference in rice grain yield (Figure 8). Moreover, Akhtar et al. (2018), Iqbal et al. (2019) and Iqbal et al. (2020) reported that changes in crop yields are strongly allied with soil biogeochemical properties and microbial biomass production.

### Relationships Between Bacterial Communities and Soil Biochemical Traits

Biochar application in combination with N fertilizer can cause physiochemical changes in soil, which can lead to changes in the composition of the bacterial community (Li et al., 2019). In the present study, we observed that biochar amendment considerably influenced soil quality traits as shown in Table 2. Furthermore, it is also reported that soil quality traits were positively correlated with the structure and composition of the bacterial community (Wu et al., 2020). Figure 8 showed the relationship of bacterial community at phylum level and soil traits including soil pH, TN, SOC, MBN, and MBC for different biochar and N rates. In our findings, RDA showed that biochar amendments in combination with N fertilizer had substantial effects on soil bacterial community and soil quality indicators compared with control (Figure 7). The dominant bacteria at the phylum level, i.e., Proteobacteria, Chloroflexi, and Acidobacteria, were positively correlated with soil properties, but the Proteobacteria were strongly associated with pH, SOC, and TN. It can be said that bacterial growth is strongly associated to the kind of fertilizer, and regulating the type and proportion of biochar amendment is an operative strategy for increasing bacterial growth. From what has been debated above, the application of biochar amendment in conjunction with reduced synthetic fertilizer may provide a faster growth environment for bacteria, thereby improving the bacterial community structure and soil fertility.

## Conclusion

The results showed that biochar amendment in combination with N fertilizer increased soil physio-biochemical properties, improved rice grain yield, increased soil bacteria, and altered fungi abundance and community structure. The bacterial Chao1, ACE, and Shannon indices were increased and fungal Chao1, ACE, and Shannon indices were decreased in biochar applied treatments as compared to sole N-applied treatments. Biochar along with N fertilizer increased number of unique OTUs of bacteria and decreased number of unique OTUs of fungi in 2020. The relationship among soil properties and soil bacteria in the combined application of biochar and N were stronger than sole N-treated soil. Furthermore, the variation in soil bacteria and fungi was closely associated with soil properties (pH, SOC, BD, TN, MBC, and MBN) and rice grain yield, which suggested that the effects of biochar in combination with N on soil bacteria and fungi community were ultimately driven by the changes in soil chemical and physical properties. These results aimed to provide a reference and basic understanding for paddy soil improvement by combined application of biochar and N with good application prospects.

## Data Availability Statement

The datasets presented in this study can be found in the NCBI under accession number PRJNA797522.

## Author Contributions

IA and LJ designed the study and wrote the manuscript. PY, IA, AI, Imran, HL, AK, SU, QZ, and MG performed the data analysis and revised the manuscript. HZ, SW, XW, AK, and QZ performed the data curation. All authors approved the submission.

## Conflict of Interest

The authors declare that the research was conducted in the absence of any commercial or financial relationships that could be construed as a potential conflict of interest.

## Publisher’s Note

All claims expressed in this article are solely those of the authors and do not necessarily represent those of their affiliated organizations, or those of the publisher, the editors and the reviewers. Any product that may be evaluated in this article, or claim that may be made by its manufacturer, is not guaranteed or endorsed by the publisher.

## Footnotes

1
www.majorbio.com
