# Supplementary material for: Biochar Amendment and Nitrogen Fertilizer Contribute to the Changes in Soil Properties and Microbial Communities in a Paddy Field
Source: Front Microbiol. 2022 Mar 23;13:834751. doi: 10.3389/fmicb.2022.834751 (PMC8984124; doi:10.3389/fmicb.2022.834751)
Supplement: Supplementary file 1 [file Data_Sheet_1.pdf]

Table 1. The number of sequences at different treatments of biochar and nitrogen fertilizers.

**Treatments**	Number of reads (Bacteria)		Number of reads (Fungi)	
	**2019**	**2020**	**2019**	**2020**
**T1**	166524b	37016.3a	63389a	20526d
**T2**	164062b	32030.6c	55090a	18048a
**T3**	147079c	26674e	45518b	14792c
**T4**	167071a	25785.7d	44140b	14361c
**T5**	177202a	28705.5d	49331b	16263c
**T6**	160013b	35227.6b	60321a	19026a
**T7**	177217a	37832.6a	42920b	14046c
**T8**	159629c	31389.3c	53588a	17676b

Note: T1=0 t B + N135 kg ha^{1}, T2= 0 t B + N180 kg ha^{1}, T3= 10 t B + N135 kg ha^{-1}, T4= 20 t B + N135 kg ha-1, T5= 30 t B + N135 kg ha^{1}, T6= 10 t B + N180 kg ha^{1}, T7= 20 t B + N180 kg ha^{-1}, T8= 30 t B + N180 kg ha^{-1}. Letters within the same column indicates statistical significance at the ($P < 0.05$).

Table 2. The abundance and diversity of OTUs from soil samples where biochar and nitrogen were applied.

2019	Chao 1 Index	ACE Index	Shannon index	Simpson Index	Chao 1 Index	ACE Index	Shannon index	Simpson Index
Treatment	Fungal (ITS1)A				Bacterial 16SR-RNA[1]			
**2019**								
T1	186.33b	185.41ab	3.11b	0.91a	15558.10a	2285.2ab	7.26a	1a
T2	160.54c	160.69b	2.69c	0.81b	15247.10a	2299.0a	7.28a	1a
T3	169.12c	169.03b	2.03e	0.75c	14868.20b	2292.0a	7.13b	1a
T4	190.36a	189.05a	2.70c	0.84b	15459.00a	2270.5c	7.24a	1a
T5	187.34b	186.69ab	3.64a	0.93a	15022.30a	2270.5c	7.15b	1a
T6	151.05d	151.4c	2.28d	0.71	14843.80b	2205.9e	7.06b	1a
T7	167.14c	167.5b	3.55a	0.92b	13678.00c	2164.5d	6.88c	0.99a
T8	181.01c	180.78a	2.16e	0.6d	14612.80c	2270.6c	7.17b	1a
**2020**								
T1	953.12a	948.21ab	5.87c	0.99a	1466.8	1417.9d	5.97a	0.99a
T2	920.3c	911.89e	5.69d	0.99a	1425.67	1459.3a	6.15ab	0.99a
T3	930.22b	927.38d	5.73c	0.99a	1454.24	1461.5a	6.19a	0.99a
T4	953.28a	947.85ab	5.90a	0.99a	1443.98	1442.1b	6.19a	0.99a
T5	956.51a	951.1a	5.89b	0.99a	1467.19	1442.1b	6.13a	0.99a
T6	959.74a	953.9a	5.78c	0.99a	1459.06	1423.8c	6.07ab	0.99a
T7	936.16b	936.78c	5.82b	0.99a	1452.7	1417.0d	6.1ab	0.99a
T8	950.27a	946.08ab	5.97a	0.99a	1452.15	1454.8a	6.15a	0.99a

Note: T1=0 t B + N135 kg ha^{1}, T2= 0 t B + N180 kg ha^{1}, T3= 10 t B + N135 kg ha^{-1}, T4= 20 t B + N135 kg ha-1, T5= 30 t B + N135 kg ha^{1}, T6= 10 t B + N180 kg ha^{1}, T7= 20 t B + N180 kg ha^{-1}, T8= 30 t B + N180 kg ha^{-1}. Letters within the same column indicates statistical significance at the ($P < 0.05$).

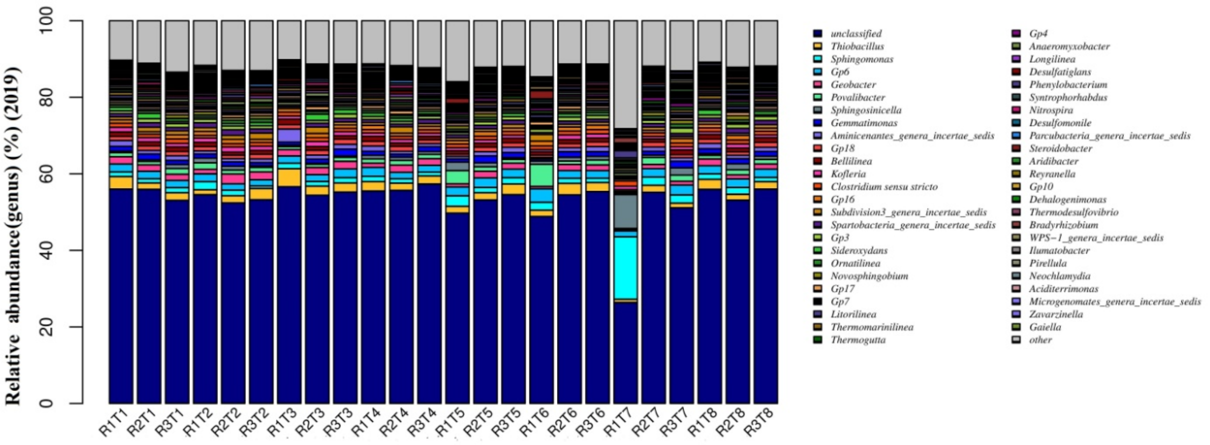

Figure 1. Changes in relative abundance on bacterial species on genus level in response to different treatments replications **(2019).** Distinct colored columns represent different species, and the length of the columns represents the species' proportion. Note: T1=0 t B + N135 kg ha^{1}, T2= 0 t B + N180 kg ha^{1}, T3= 10 t B + N135 kg ha^{-1}, T4= 20 t B + N135 kg ha-1, T5= 30 t B + N135 kg ha^{1}, T6= 10 t B + N180 kg ha^{1}, T7= 20 t B + N180 kg ha^{-1}, T8= 30 t B + N180 kg ha^{-1}.

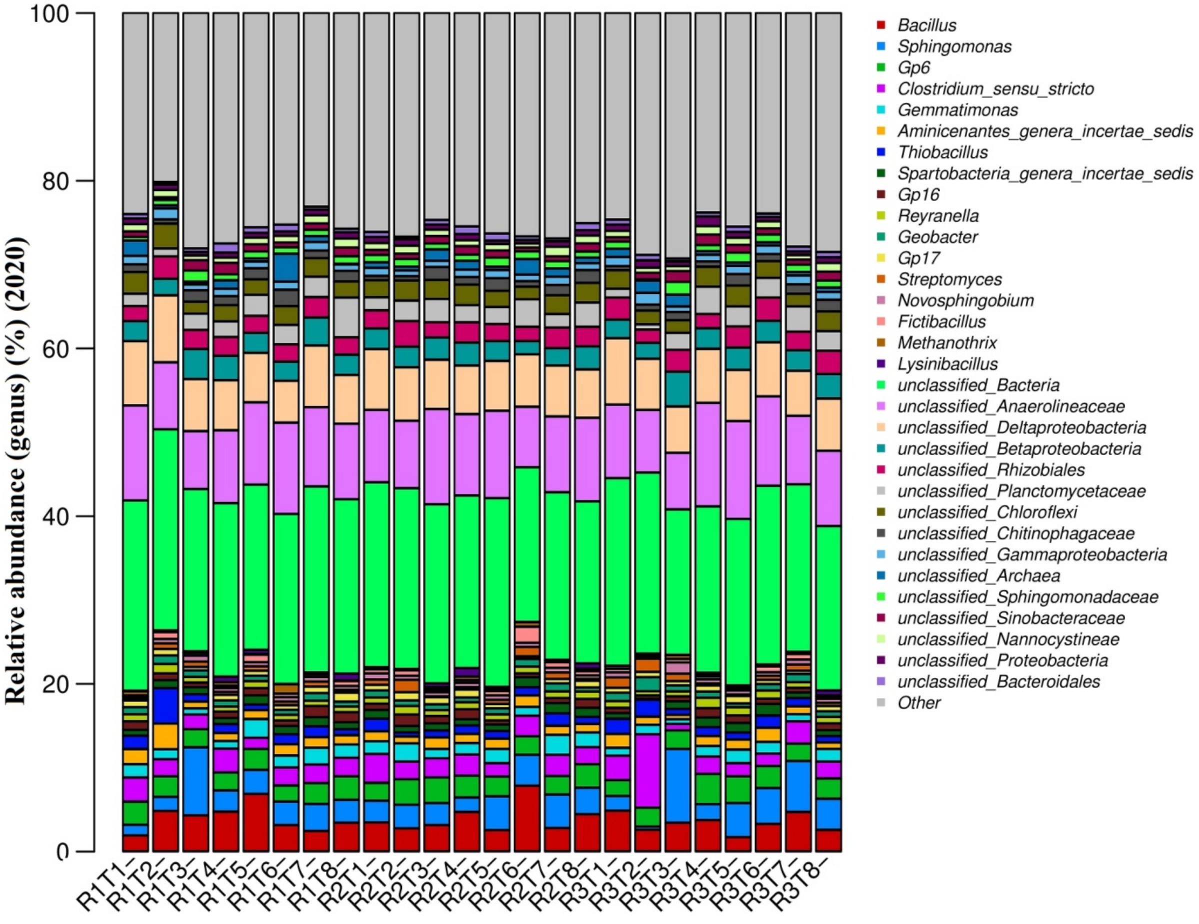

Figure 2. Changes in relative abundance on bacterial species on genus level in response to different treatments replications **(2020).** Distinct colored columns represent different species, and the length of the columns represents the species' proportion. Note: T1=0 t B + N135 kg ha^{1}, T2= 0 t B + N180 kg ha^{1}, T3= 10 t B + N135 kg ha^{-1}, T4= 20 t B + N135 kg ha-1, T5= 30 t B + N135 kg ha^{1}, T6= 10 t B + N180 kg ha^{1}, T7= 20 t B + N180 kg ha^{-1}, T8= 30 t B + N180 kg ha^{-1}.

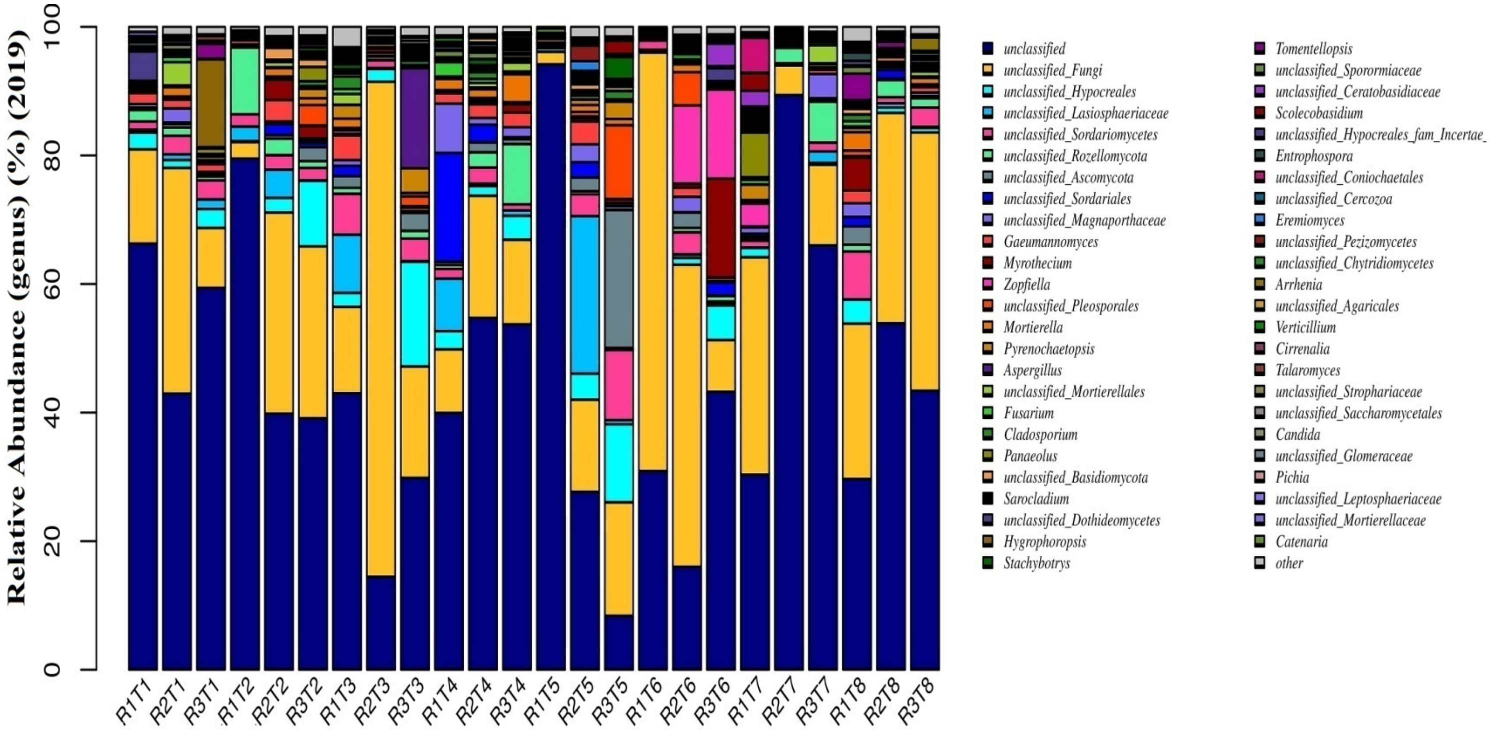

Figure 3. Changes in relative abundance on fungal species on genus level in response to different treatments replications (2019). Distinct colored columns represent different species, and the length of the columns represents the species' proportion. Note: T1=0 t B + N135 kg ha^{1}, T2= 0 t B + N180 kg ha^{1}, T3= 10 t B + N135 kg ha^{-1}, T4= 20 t B + N135 kg ha-1, T5= 30 t B + N135 kg ha^{1}, T6= 10 t B + N180 kg ha^{1}, T7= 20 t B + N180 kg ha^{-1}, T8= 30 t B + N180 kg ha^{-1}.

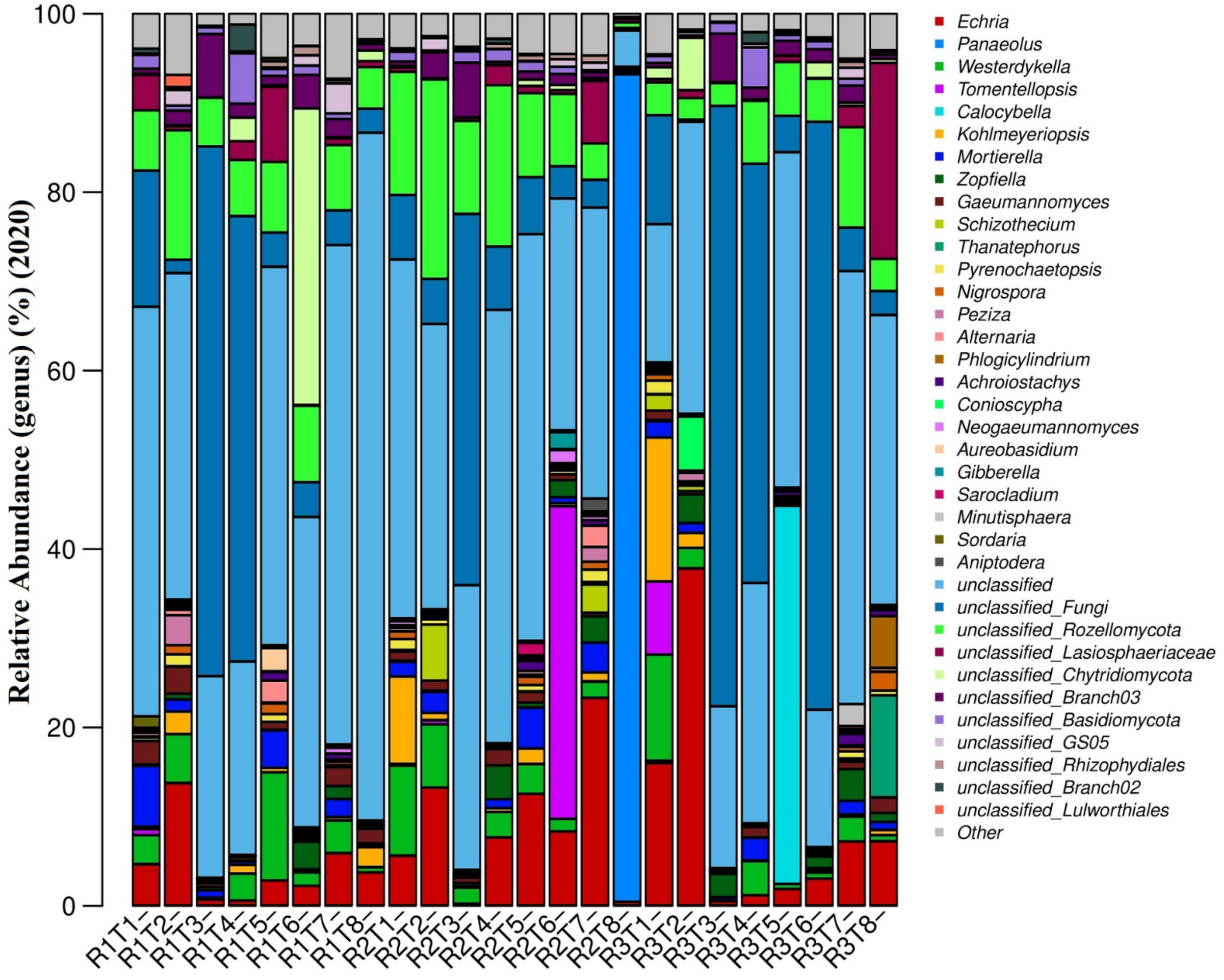

Figure 4. Changes in relative abundance on fungal species on genus level in response to different treatments replications (2020). Distinct colored columns represent different species, and the length of the columns represents the species' proportion. Note: T1=0 t B + N135 kg ha^{1}, T2= 0 t B + N180 kg ha^{1}, T3= 10 t B + N135 kg ha^{-1}, T4= 20 t B + N135 kg ha-1, T5= 30 t B + N135 kg ha^{1}, T6= 10 t B + N180 kg ha^{1}, T7= 20 t B + N180 kg ha^{-1}, T8= 30 t B + N180 kg ha^{-1}.

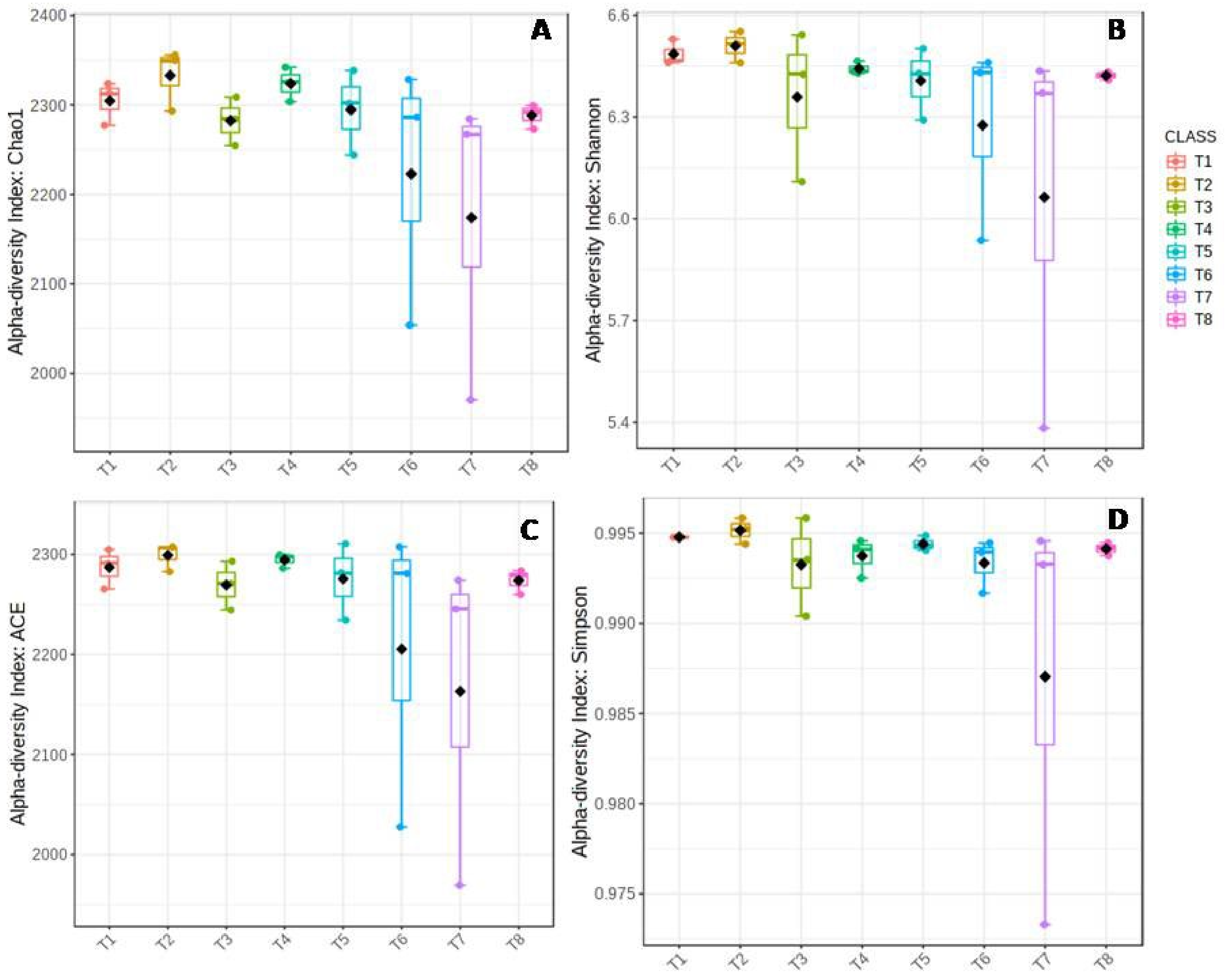

Figure 5. The abundance and diversity of OTUs from soil samples in 2019, where biochar and nitrogen were applied. Simpson: (p-value: 0.43982; [ANOVA] F-value: 1.0447) Chao 1: p-value: 0.36813; [ANOVA] F-value: 1.1779), ACE: (p-value: 0.45623; [ANOVA] F-value: 1.0167) Shanon: (p-value: 0.46918; [ANOVA] F-value: 0.99521). Note: T1=0 t B + N135 kg ha^{1}, T2= 0 t B + N180 kg ha^{1}, T3= 10 t B + N135 kg ha^{-1}, T4= 20 t B + N135 kg ha-1, T5= 30 t B + N135 kg ha^{1}, T6= 10 t B + N180 kg ha^{1}, T7= 20 t B + N180 kg ha^{-1}, T8= 30 t B + N180 kg ha^{-1}.

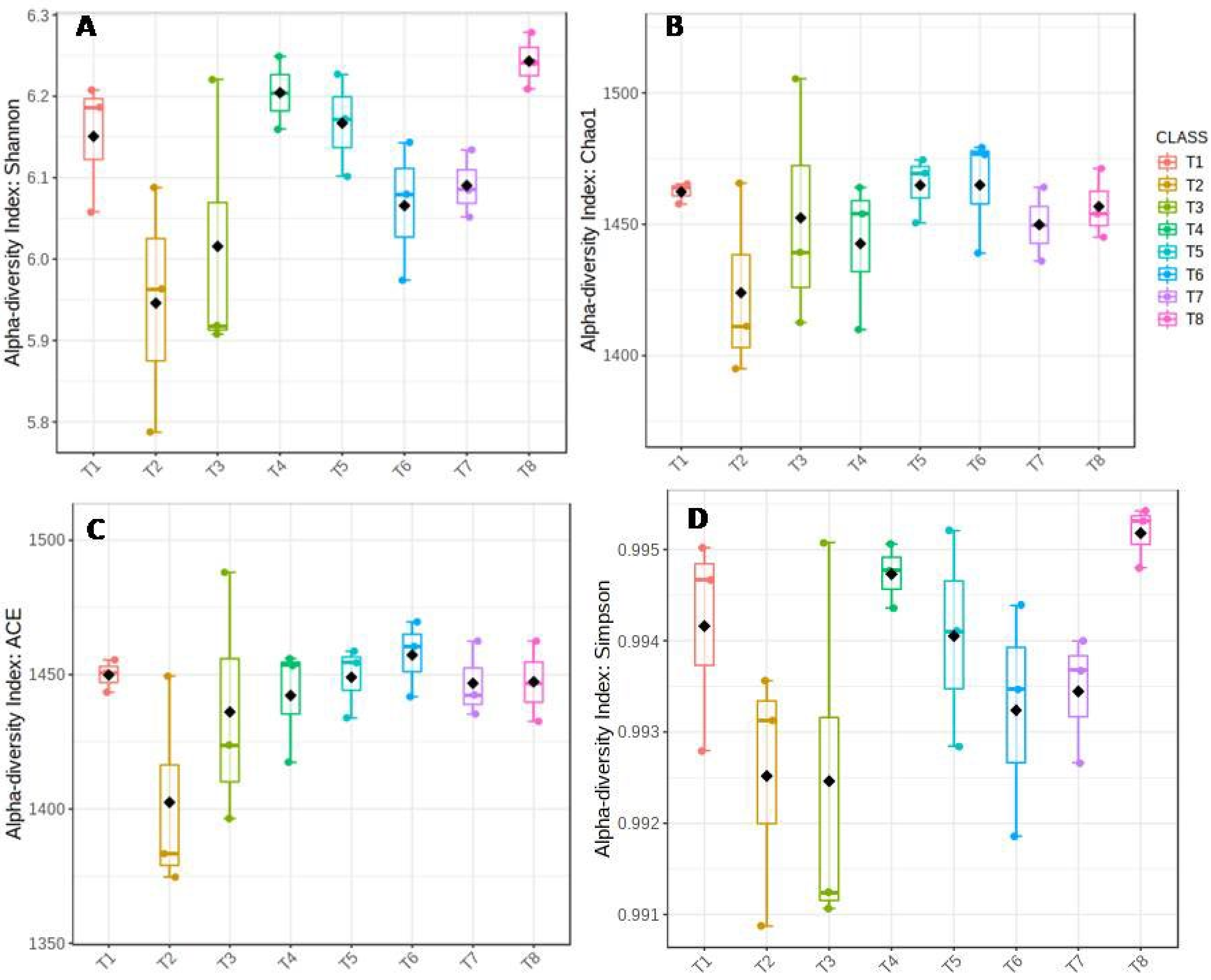

Figure 6. The abundance and diversity of OTUs from soil samples in 2020, where biochar and nitrogen were applied. Simpson: (p-value: 0.14041; [ANOVA] F-value: 1.8779), Chao 1: (p-value: 0.5773; [ANOVA] F-value: 0.8305), ACE: (p-value: 0.30493; [ANOVA] F-value: 1.3161), Shanon: (p-value: 0.028799; [ANOVA] F-value: 3.1016). Note: T1=0 t B + N135 kg ha^{1}, T2= 0 t B + N180 kg ha^{1}, T3= 10 t B + N135 kg ha^{-1}, T4= 20 t B + N135 kg ha-1, T5= 30 t B + N135 kg ha^{1}, T6= 10 t B + N180 kg ha^{1}, T7= 20 t B + N180 kg ha^{-1}, T8= 30 t B + N180 kg ha^{-1}.

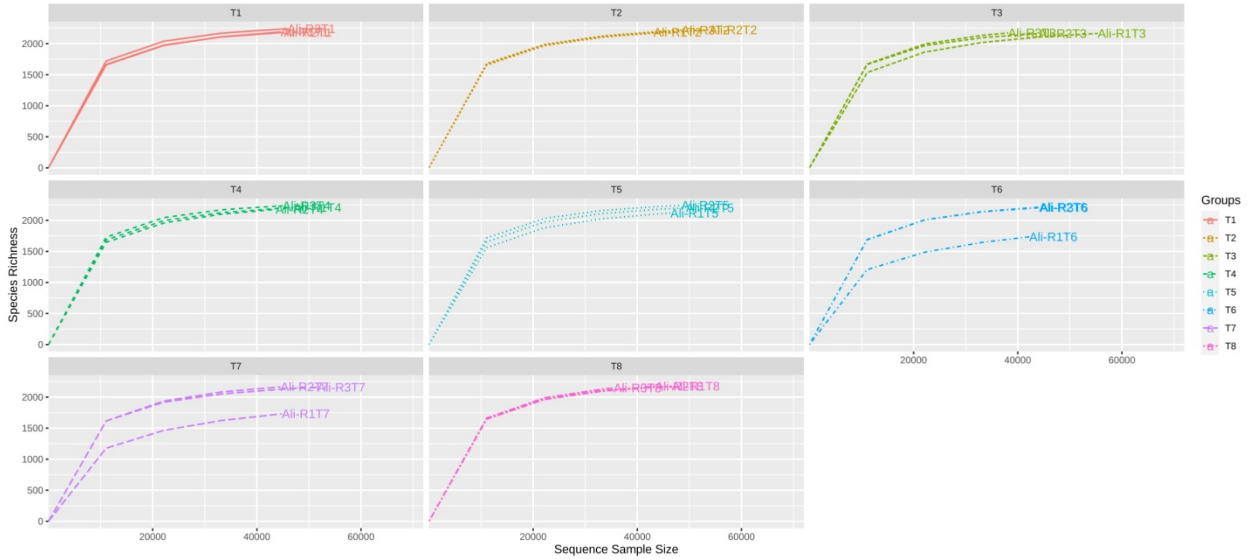

Figure 7. Rarefaction curves of 16S rRNA sequencing depth and number of bacterial species numbers in soil depth (0-20cm) in 2019 . Note: T1=0 t B + N135 kg ha^{1}, T2= 0 t B + N180 kg ha^{1}, T3= 10 t B + N135 kg ha^{-1}, T4= 20 t B + N135 kg ha-1, T5= 30 t B + N135 kg ha^{1}, T6= 10 t B + N180 kg ha^{1}, T7= 20 t B + N180 kg ha^{-1}, T8= 30 t B + N180 kg ha^{-1}.

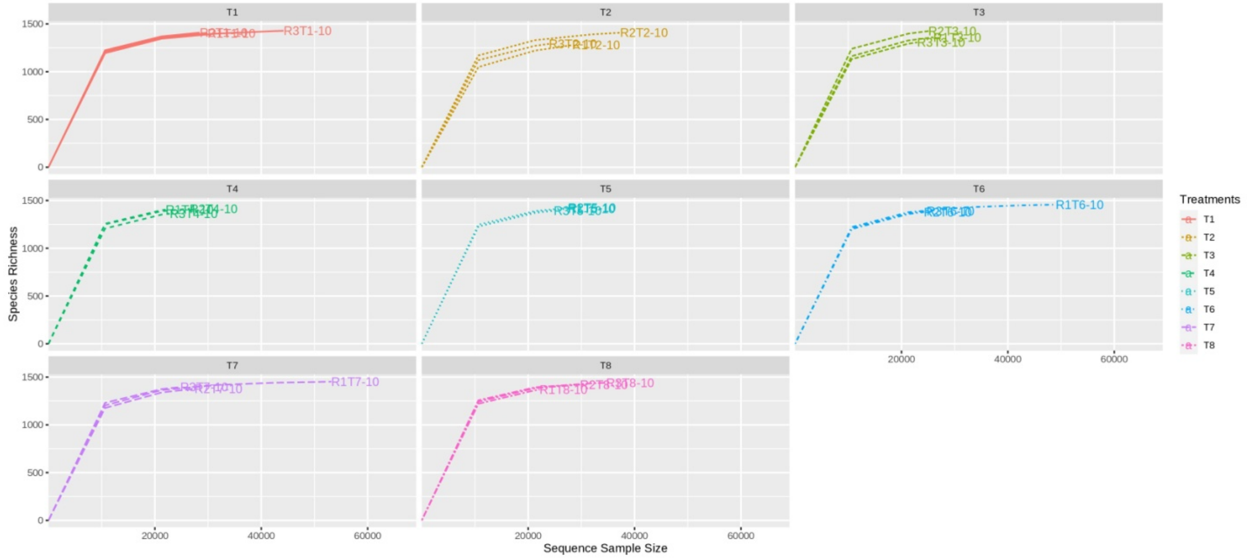

Figure 8. Rarefaction curves of 16S rRNA sequencing depth and number of bacterial species numbers in soil depth (0-20cm) in 2020. Note: T1=0 t B + N135 kg ha^{1}, T2= 0 t B + N180 kg ha^{1}, T3= 10 t B + N135 kg ha^{-1}, T4= 20 t B + N135 kg ha-1, T5= 30 t B + N135 kg ha^{1}, T6= 10 t B + N180 kg ha^{1}, T7= 20 t B + N180 kg ha^{-1}, T8= 30 t B + N180 kg ha^{-1}.

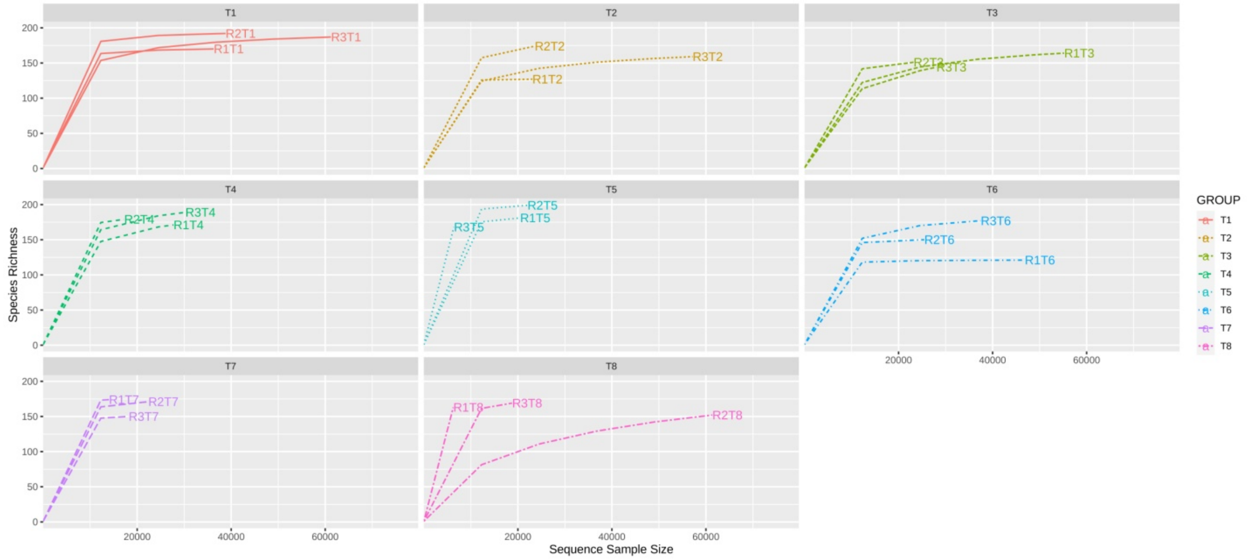

Figure 9. Rarefaction curves of 16S rRNA sequencing depth and number of fungal species numbers in soil depth (0-20cm) in 2019. Note: T1=0 t B + N135 kg ha^{1}, T2= 0 t B + N180 kg ha^{1}, T3= 10 t B + N135 kg ha^{-1}, T4= 20 t B + N135 kg ha-1, T5= 30 t B + N135 kg ha^{1}, T6= 10 t B + N180 kg ha^{1}, T7= 20 t B + N180 kg ha^{-1}, T8= 30 t B + N180 kg ha^{-1}.

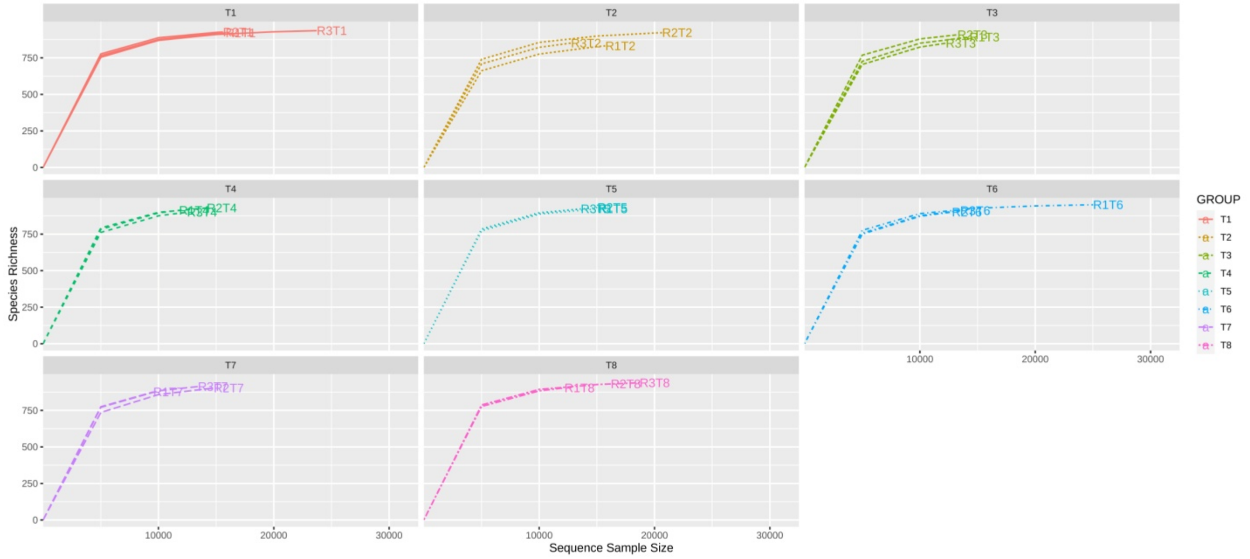

Figure 10. Rarefaction curves of 16S rRNA sequencing depth and number of fungal species numbers in soil depth (0-20cm) in 2020. Note: T1=0 t B + N135 kg ha^{1}, T2= 0 t B + N180 kg ha^{1}, T3= 10 t B + N135 kg ha^{-1}, T4= 20 t B + N135 kg ha-1, T5= 30 t B + N135 kg ha^{1}, T6= 10 t B + N180 kg ha^{1}, T7= 20 t B + N180 kg ha^{-1}, T8= 30 t B + N180 kg ha^{-1}.
